# Real-Time Monitoring and Evaluation of a Visual-Based Cervical Cancer Screening Program Using a Decision Support Job Aid

**DOI:** 10.3390/diagnostics6020020

**Published:** 2016-05-16

**Authors:** Curtis W. Peterson, Donny Rose, Jonah Mink, David Levitz

**Affiliations:** 1 MobileODT, Ltd., Tel Aviv 67107, Israel; curtispeterson@mobileodt.com (C.P.); donnyrose@mobileodt.com (D.R.); jonahmink@mobileodt.com (J.M.); 2 Department of Family Medicine and Community Health, University of Pennsylvania Health System, Philadelphia, PA 19104, USA

**Keywords:** cervical cancer, screening, mHealth, low-resource settings, visual inspection with acetic acid, VIA

## Abstract

In many developing nations, cervical cancer screening is done by visual inspection with acetic acid (VIA). Monitoring and evaluation (M&E) of such screening programs is challenging. An enhanced visual assessment (EVA) system was developed to augment VIA procedures in low-resource settings. The EVA System consists of a mobile colposcope built around a smartphone, and an online image portal for storing and annotating images. A smartphone app is used to control the mobile colposcope, and upload pictures to the image portal. In this paper, a new app feature that documents clinical decisions using an integrated job aid was deployed in a cervical cancer screening camp in Kenya. Six organizations conducting VIA used the EVA System to screen 824 patients over the course of a week, and providers recorded their diagnoses and treatments in the application. Real-time aggregated statistics were broadcast on a public website. Screening organizations were able to assess the number of patients screened, alongside treatment rates, and the patients who tested positive and required treatment in real time, which allowed them to make adjustments as needed. The real-time M&E enabled by “smart” diagnostic medical devices holds promise for broader use in screening programs in low-resource settings.

## 1. Introduction

Cervical cancer is the leading cause of cancer death for women in low-resource settings, with 528,000 new cases each year that result in 266,000 deaths [1]. In contrast to low- and middle-income countries (LMICs), cervical cancer rates in member countries of the Organization for Economic Cooperation and Development (OECD) have declined drastically since the implementation of regular Pap screening 50 years ago [2]. However, Pap testing requires a clinical workforce and laboratory infrastructure that is lacking in many LMICs, and as a result, patients do not have access to this level of regular screening. It is widely believed that increasing access to regular cervical cancer screening in LMICs would reduce mortality from the disease [1,3].

In 70 LMICs around the world, cervical cancer screening is done by visual inspection with acetic acid (VIA), an easy to implement procedure with limited diagnostic accuracy in which a practitioner applies a thin layer of 3%–5% acetic acid to the cervix and visualizes it from outside the vaginal canal using a light source [4]. Previous studies have shown that VIA is a cost-effective screening method for cervical cancer in LMICs [5], but its contribution to improving health outcomes is difficult to estimate [6]. The low cost ($6 per test) and relative ease of implementation of VIA programs make it an appealing option for many public health programs, particularly when coupled with onsite treatment of suspicious lesions with cryotherapy ($28 per application) [7]. However, VIA suffers from a positive predictive value (PPV) of only 17%, meaning approximately five of six positive diagnoses are actually false [4]. This leads to overtreatment, which inflicts unnecessary pain on many patients, and costs under-resourced health systems money, equipment, and time.

Despite lower diagnostic accuracy than other screening methods, the World Health Organization recommends VIA for low-resource settings in which Pap- or HPV-based screening programs are unfeasible [8]. Following these guidelines, governments and non-governmental organizations (NGOs) have established and supported VIA programs [9,10,11]. For many of these VIA programs, comprehensive monitoring and evaluation (M&E) at the health systems level is challenging for several reasons. First, patient data and resource records are mostly paper-based. Second, assessing screener competency is logistically difficult, since it requires more experienced practitioners to review cases with screeners at the point-of-care. Often VIA programs, which operate in resource-constrained environments, lack basic yet important records on the patient population, rates of different outcomes, and even the program’s medical supplies inventory.

**Figure 1:**
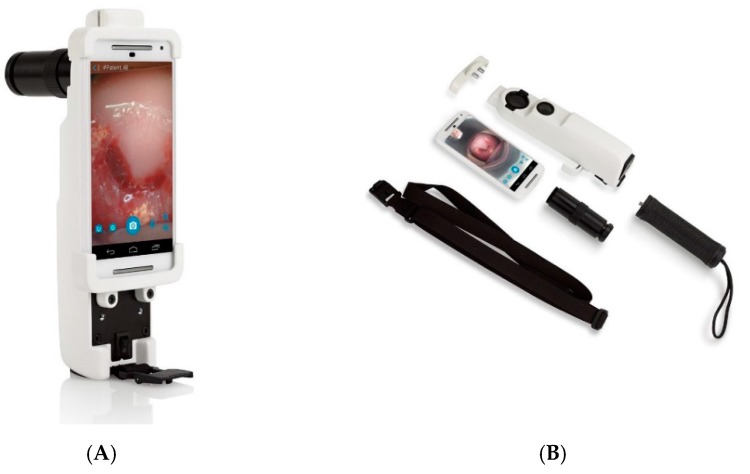
Photos of the Enhanced Visual Assessment (EVA) System’s mobile colposcope, either assembled (**A**), or split into its various components (**B**).

To better address the needs of VIA programs, the Enhanced Visual Assessment (EVA) System was developed by MobileODT (Tel Aviv, Israel). The EVA System augments the VIA procedure in two main ways. On the hardware side, it provides magnification and a reliable, appropriate light source for visualization, while the software allows for real-time workflow support and procedure logging for monitoring and evaluation. The system consists of a low-cost mobile colposcope built around a smartphone (Figure 1), and an online image portal for access and review of patient data. The mobile colposcope consists of a medical-grade plastic case that houses a smartphone, and a white light LED with a battery pack for illumination. Orthogonal polarizers on the illumination and detection ends are used to reduce glare in the images. Light reflected off the cervix forms an image on the smartphone camera, which is controlled by an app that was designed to be HIPAA-compliant. The app relays cervical images to the online database for storage and documentation. Both optical and digital zooms are possible. The EVA System was designed for use by both doctors and nurses, who perform the majority of VIA screening procedures.

The mobile colposcope app, CervDx, allows for increased flexibility in using EVA, as software updates can be used to rapidly implement and test new features. One feature of particular interest to many VIA programs is a job aid that documents the diagnosis and treatment decisions of the nurse that conducted VIA screening. The job aid is a software feature that guides provider workflow and records data automatically. This feature was designed to improve M&E in existing VIA programs. 

In this paper, we tested the implementation of the novel job aid feature on the mobile colposcope app during an EVA deployment in a cervical cancer screening camp in Nairobi, Kenya. We show that the integration of this feature allows for improved M&E of VIA programs by providing key statistics in real time.

## 2. Materials and Methods

### 2.1. Cervical Cancer Screening Camp

**Figure 2:**
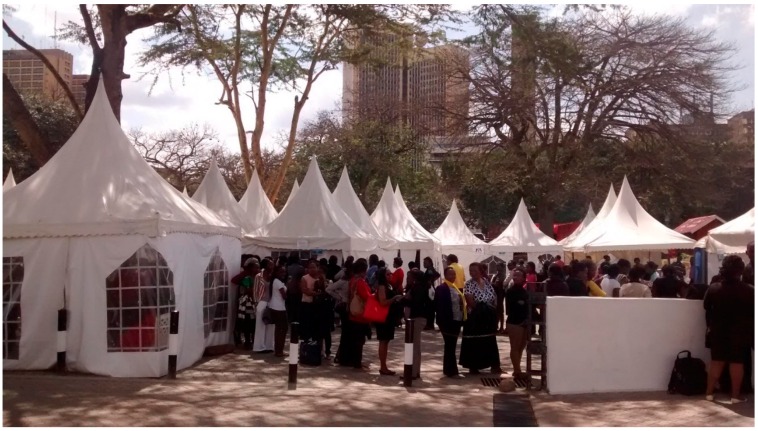
Screening camp at the 9th “Stop Cervical, Breast and Prostate Cancer in Africa“ (SCCA) conference offering free cervical cancer screening.

Cervical cancer is a significant problem in Kenya, where it is estimated that in 2014, 2451 patients died from the disease [12]. In order to raise awareness of cervical cancer in Africa, the First Lady of Kenya hosted the 9th “Stop Cervical, Breast and Prostate Cancer in Africa” (SCCA) conference in Nairobi in July 2015. To promote the importance of cancer screening, the Kenyan Ministry of Health, along with non-governmental organizations (NGOs) and private sector companies, organized a weeklong screening camp as part of the SCCA conference. For seven days, anyone could come and be screened for cervical cancer (Figure 2). In addition, screening for breast cancer, prostate cancer, and HIV testing and counseling services were also offered.

Six (*N* = 6) organizations conducted cervical cancer screening using the EVA system at the SCCA screening camps: the Health Ministry of Nairobi City County, Aga Kahn University Hospital, Family Health Options Kenya, Johns Hopkins International Program in Gynecology and Obstetrics, Kenya, SOS Children’s Villages, Kenya, and Population Services, Kenya. Each organization was allotted a group of tents for the purpose of screening (Figure 2). Every participating organization agreed to conduct visual cervical screening at the camp using EVA with the integrated job aid for decision support and data capture.

### 2.2. EVA Deployment

Altogether, seventeen EVA System devices were deployed to the various organizations at the SCCA screening camp. As part of the deployed infrastructure, SIM cards were installed in device phones, to ensure that the cervical images taken with the mobile colposcope uploaded to the image portal. Diagnostic and treatment results from the organizations were compiled together on the back end of the portal and displayed on monitors throughout the conference in real-time.

#### 2.2.1. EVA Training

Prior to the SCCA screening camp, the nurses that conduct the visual screening for the various organizations were trained on using the EVA System, including the app. Training was conducted the morning that the screening camp began, and emphasized the standard VIA workflow, and how EVA can be used to augment the procedure, including the job aid. Trainers remained onsite throughout the camp to help troubleshoot any technical issues and provide ongoing guidance on use of the technology.

#### 2.2.2. Clinical Workflow Using EVA 

In routine VIA procedures, a speculum is inserted into the vagina to open up the vaginal canal and enable visualization of the cervix. A thin layer of acetic acid is applied using an elongated swab, which removes moisture from the cells and turns areas with cervical dysplasia white over approximately 2 min (the acetowhitening process is also used in colposcopy procedures in higher level clinics). The cervix is illuminated from outside the patient’s body and visualized by the clinician. If the clinician observes white area(s) on the cervix, the test result is considered positive.

**Figure 3:**
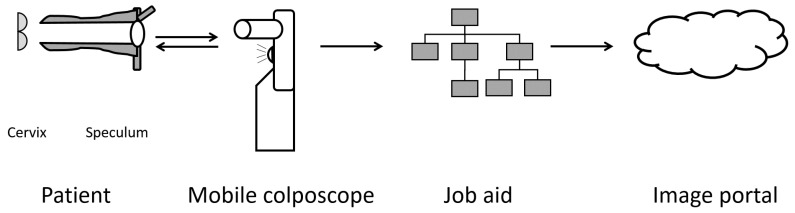
Visual inspection with acetic acid (VIA) clinical workflow with the EVA System. Light from the mobile colposcope illuminates the cervix (through the opening of a speculum). The nurse records images and makes a clinical decision using the guided workflow job aid. The images and clinical decision are uploaded to the cloud-based image portal.

The clinical workflow in VIA procedures augmented by EVA (Figure 3) is nearly identical, except that the cervix is visualized using the mobile colposcope that is placed 10–15 cm from the vaginal introitus. Two minutes after the application of acetic acid, the clinician (nurse) visualizes and records images of the cervix using the mobile colposcope. Following the exam, the nurse shows the patient an image of her cervix, records a clinical decision, and instructs the patient accordingly. The image and decision are uploaded to the secure, web-based image portal.

#### 2.2.3. Decision Support Job Aid

**Figure 4:**
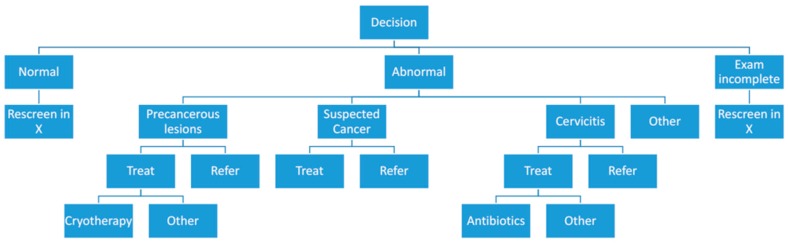
A schematic of the decision support job aid.

The key feature under investigation in this paper is the decision support job aid, which was integrated into the EVA System mobile application and used by the nurses to document their clinical decisions. A graphical description of the workflow provided by the job aid is shown in Figure 4. It was designed to account for various clinical results from VIA procedures: Normal/abnormal cervix, where abnormal results are further categorized as pre-cancerous lesion, suspected cancer, cervicitis, or other. Treatment options and referrals were also included. Using the job aid added approximately two minutes to the screening procedure. Results of the diagnosis and treatment decisions were uploaded onto the web-based image portal and compiled. Statistics on the number of patients screened, clinical findings, and treatments were calculated for each participating organization, and aggregated data from the entire SCCA camp were made publicly available.

## 3. Results

### 3.1. Aggregated SCCA Screening Camp Results

**Figure 5:**
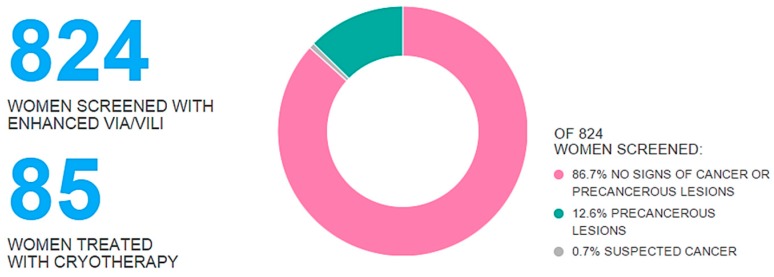
Aggregated screening and treatment data from the entire SCCA screening camp. The graphic is a screenshot from http://savelives.today/sccakenya2015.

Statistics from VIA screening using the EVA system at the entire SCCA screening camp were aggregated and graphically displayed on the web in real time at savelives.today/sccakenya2015 (Figure 5). Monitors placed throughout the SCCA conference displayed the results on the website. Statistics updated every time a new patient case uploaded into the system. The website updated every 5 min.

### 3.2. Individual Organizational Results

In addition to the aggregated SCCA results, each participating organization was also given its compiled internal results at the end of the SCCA screening camp. These showed the total number of patient cases seen by the organization as a whole, as well as a breakdown to individual nurses. Moreover, the rates of positive test results and treatment were also shown, both by organization and by individual users. The results compared an individual organization to the screening camp as a whole, as well as compared individual nurses to their colleagues in the organization. This gives both the organizations and their nurses a glimpse of how they compare against their peers, and enables them to take corrective action to address trends of abnormal screening patterns. Note that individual organizational results were not displayed publicly, as they are considered confidential information of the respective organization.

## 4. Discussion

In this paper, 17 mobile colposcope devices were deployed to a cervical cancer screening camp in which six different organizations conducted VIA screening. We integrated a novel decision support job aid designed to improve M&E of VIA programs into the app used to control the mobile colposcope, and used it to document diagnostic and treatment decisions associated with captured images. The results from the job aid were compiled in an online database and aggregate screening statistics were displayed in real time, both on the web and at the SCCA screening camp and conference, presenting participating organizations with a live snapshot of the extent of their screening efforts. This implementation of the decision support job aid represents, to our knowledge, the first real-time M&E of a VIA screening program in a low-resource setting.

The implementation of the decision support job aid into the EVA System exemplifies a model of integrating connected devices at the point-of-care to rapidly yield a large body of clinical and programmatic data. Within one week, clinical decisions from over 800 patient cases were recorded and classified by clinical decision, treatment provided, organization, and user. This volume of data can be further analyzed to better assess the prevalence/impact of cervical dysplasia and cervical cancer in regions for which, until now, such information did not exist. At the health systems level, this information can better inform policy decisions, and therefore holds considerable promise for improving public health outcomes.

Likewise, the information collected by the decision support job aid has important implications for VIA screening programs. In many traditional VIA screening programs, paper records are often not properly kept, as they are not fully integrated into the clinical workflow. By integrating the decision support job aid into the app controlling the mobile colposcope, organizations were able to leverage technology to improve their record keeping without adding numerous steps outside of screeners’ clinical workflow. They were also able to compare themselves against their peers. For example, one participating organization’s treatment rate recorded was above 50%, relative to the SCCA average of 12.6%. Further inquiry into the organization’s practice showed that they used the mobile colposcope as a secondary screen rather than a primary screen. This discrepancy illustrated how differences in programmatic implementation result in different clinical data, which was discovered while the screening camp was still running.

Similarly, for organizational M&E at the practitioner level, the decision support job aid enabled deeper analysis into the clinical decision making patterns of nurses. For example, more experienced nurses frequently had a lower treatment rate than less experienced nurses. Some of the organizations were able to implement corrective actions in real time based on real M&E data, which they claimed improved the quality of care. Additionally, they could also implement more targeted continuing medical education modules for their nurses based on competency level informed by the gathered statistics.

In addition to the improved M&E metrics enabled by the decision support job aid, several surprising results of visual cervical cancer screening using the EVA System were observed. One such outcome was that nurse confidence was greatly improved as a result of the EVA System. Because they had a digital image recorded with a clinical decision, many nurses felt empowered by the device. It offered them a way to continually educate themselves by reviewing cases and consulting with colleagues. Until now, many nurses claimed they did not practice VIA frequently enough to feel comfortable with the procedure. This lack of practitioner confidence is likely to be correlated to the poor accuracy of VIA [4]. With the EVA System, the interaction with the patient was also improved, as now nurses were able to show patients an image of their cervix and have more informed discussions about treatment if necessary. If deployed on a large scale, the EVA System may prove to be usefully integrated in training courses (both basic and advanced) for the nurses and operators at large involved in performing VIA.

It is important to note that the implementation of the decision support job aid presented in this paper does not represent a controlled trial in which one group is compared against another. Rather, the data shown here can be considered as a hybrid of a proof-of-concept experiment and a case study. Implementing such a controlled trial is challenging. Most patients refused to be screened by the naked eye once they were aware the EVA System was being used by others. One nurse epitomized this trend, asking to be screened herself once she saw the EVA System in practice. Until that point, she was afraid of the VIA procedure herself, despite having conducted it hundreds of times. Such qualitative evidence of patient psychology is not well documented, particularly for procedures such as VIA conducted in low-resource settings. Unfortunately, financial constraints keep such research from taking place, despite its importance to patient well-being.

The proof-of-concept deployment of the decision support job aid in a cervical cancer screening camp had several challenges. Each of the six organizations implemented their EVA-augmented VIA procedures slightly differently, and so it was difficult to ensure a fair comparison between programs. Also, providing proper connectivity throughout the camp at any given moment was not always feasible. Increasing patient awareness about cervical cancer screening and the camp was also problematic, and many patients only showed up on the last day. (In fact, many of the organizers of the screening camp expected a much larger patient turn out, and the EVA System M&E data actually showed the limitations of their outreach efforts.) Future technological refinements will focus on addressing these challenges. Specifically, new version of the app will be designed to ensure data upload in conditions of fragile connectivity. Additionally, new efforts will address standardizing the implementation of VIA with EVA across screening partners.

## 5. Conclusions

The EVA System was deployed to a cervical cancer screening camp in Kenya, with an added feature that monitored the clinical decisions of the nurses practicing the VIA procedure. The implementation of the decision support job aid, coupled with integration on the back end, enabled real time M&E of the VIA screening program. Successful implementations of connected diagnostic devices holds promise for improving the quality of care at the health system, organizational, and practitioner level.

## Abbreviations

The following abbreviations are used in this manuscript:

VIA	Visual inspection with acetic acid
EVA	Enhanced visual assessment
M&E	Monitoring and evaluation
LMICs	low- and middle-income countries
OECD	Organization for Economic Cooperation and Development
